# Adverse Reactions to Phenol Neurolysis: Report of Severe Necrosis and Infection Following Genicular Nerve Ablation

**DOI:** 10.1002/ccr3.70885

**Published:** 2025-09-12

**Authors:** Michael D. Morodomi, Melissa P. Cortes, Michael Smerina, Tatjana Gavrancic, Ricardo J. Pagan, Adrian G. Dumitrascu, Aditya A. Khanijo, Nilaa Gopikrishnan, Aleksandra Murawska Baptista

**Affiliations:** ^1^ Office of Non Clinical Education Mayo Clinic Jacksonville Florida USA; ^2^ Division of Hospital Internal Medicine Mayo Clinic Jacksonville Florida USA; ^3^ Department of Critical Care Medicine Mayo Clinic Jacksonville Florida USA

**Keywords:** adverse reaction, chemical neurolysis, injection site reaction, phenol

## Abstract

This report details a severe complication of phenol neurolysis in a diabetic patient, where phenol induced tissue necrosis can lead to severe cellulitis with ulceration that may prove difficult to treat. It emphasizes the importance of considering comorbidities when selecting phenol neurolysis for treatment of chronic pain.

## 1 Introduction

Phenol is a corrosive chemical agent that readily denatures protein and can cause denervation via demyelination of axons and Wallerian degeneration [1, 2, 3]. Phenol is composed of carbolic acid, phenylic acid, phenyl hydroxide, hydroxybenzene, and oxybenzone [2]. A notable study by Nathan and Sears [4] found that phenol has two effects at different concentrations. At lower concentrations (≤ 2%), a local anesthetic effect was observed, while at higher concentrations (> 3%), axonal degeneration was noted [4]. Phenol is also said to have a bactericidal and fungicidal effect at concentrations of 6% to 10% [5].

Chemical neurolysis is commonly reserved for the treatment of chronic intractable pain that has failed to respond to conventional pain therapies, treatment of cancer‐related pain among those with advanced malignancy and short life expectancy, and for the treatment of spasticity [2]. There are currently no strict selection criteria to guide patient candidacy; however, absolute contraindications include patients with an active infection, the presence of a tumor at the needle insertion site, elevated risk of bleeding at the injection site and/or among patients who are on anticoagulation therapy, allergy to the selected chemical agent, and patient refusal [2, 6].

The treatment technique generally entails the implementation of aseptic measures and the use of ultrasound, fluoroscopy, or nerve stimulation to identify the target nerve. Once identified, the needle is then advanced until the desired nerve is reached. A nerve block with local anesthetic can be performed to confirm the correct position of the needle, and once confirmed, the phenol can subsequently be injected [2].

Phenol can be mixed in aqueous, glycerin, or lipid solutions depending on the desired effect. An aqueous solution yields a more potent neurolytic effect with a faster rate of diffusion and a broader area of spread, while a glycerin solution results in a more viscous product with a more limited area of spread [2]. There is no specific dose recommendation for phenol, although a dose concentration of 3%–12% has been suggested [2].

Cardiotoxic effects, such as cardiac arrhythmia and intraoperative cardiac arrest, during chemical splanchnicectomy with phenol in patients with pancreatic cancer have been reported [2]. Other systemic adverse effects include nausea, vomiting, bradycardia, and central nervous system stimulation that have also been mentioned [2]. Localized adverse effects that have been commonly reported include local tissue damage, dysesthesia/hyperesthesia, and infection [2, 4]. Since phenol is metabolized in the liver and excreted via the kidneys, it is typically avoided in patients with advanced hepatic disease. Chronic exposure can lead to renal toxicity, skin lesions, and gastrointestinal adverse effects. Adverse effects are uncommon if systemic doses are < 100 mg [2].

Overall, the literature on the clinical use of phenol in neurolysis is limited, and multiple studies and case reports have bolstered the safety and efficacy of its use in nerve ablation. Although adverse effects of this procedure have been noted, reports of significant complications or severe adverse reactions following phenol‐induced neurolysis procedures are rare. Our objective is to enhance understanding of these potential serious adverse outcomes by presenting a case of a 56‐year‐old female with type 2 diabetes, obesity, and osteoarthritis of the left knee who developed severe tissue necrosis, ulceration, and infection following phenol neurolysis of the genicular nerve.

## 2 Case History/Examination

A 56‐year‐old woman with a history of type 2 diabetes, morbid obesity (height, 167.6 cm; weight, 154 kg; body mass index, 55), hypertension, and osteoarthritis presented to the emergency department of another institution with left lower extremity pain. Two days before the emergency department visit, the patient received three phenol injections for neurolysis of the genicular nerve branches. The injections were performed at an outside institution, and the exact phenol dose used was not documented. The procedure was pursued for the management of chronic pain related to osteoarthritis of her left knee, which had been unresponsive to standard multimodal pain regimens and physical therapy. Prior to the procedure, the patient had also explored total knee arthroplasty as a solution to her chronic knee pain and was deemed of poor candidacy due to her comorbid conditions.

Upon examination, the patient's left leg displayed localized swelling, erythema, and tenderness. The injection site was dark in appearance with surrounding erythema on the frontal aspect of her left leg just below the knee. The area was warm to the touch without evidence of skin breakdown. Vital signs and laboratory workup were within normal limits.

## 3 Methods (Differential Diagnosis, Investigation and Treatment)

The initial workup included imaging to assess for deep tissue infection and venous thromboembolism. Computed tomography showed a small suprapatellar joint effusion without evidence of deep tissue infection or fracture. Venous ultrasound was negative for superficial or deep venous thromboembolism. The patient received a single dose of ceftriaxone 1 g/day due to concern for possible cellulitis. Ultimately, the pain, swelling, and erythema were attributed to a phenol‐induced chemical burn. Antibiotics were discontinued, and the patient was discharged home with analgesics.

**FIGURE 1:**
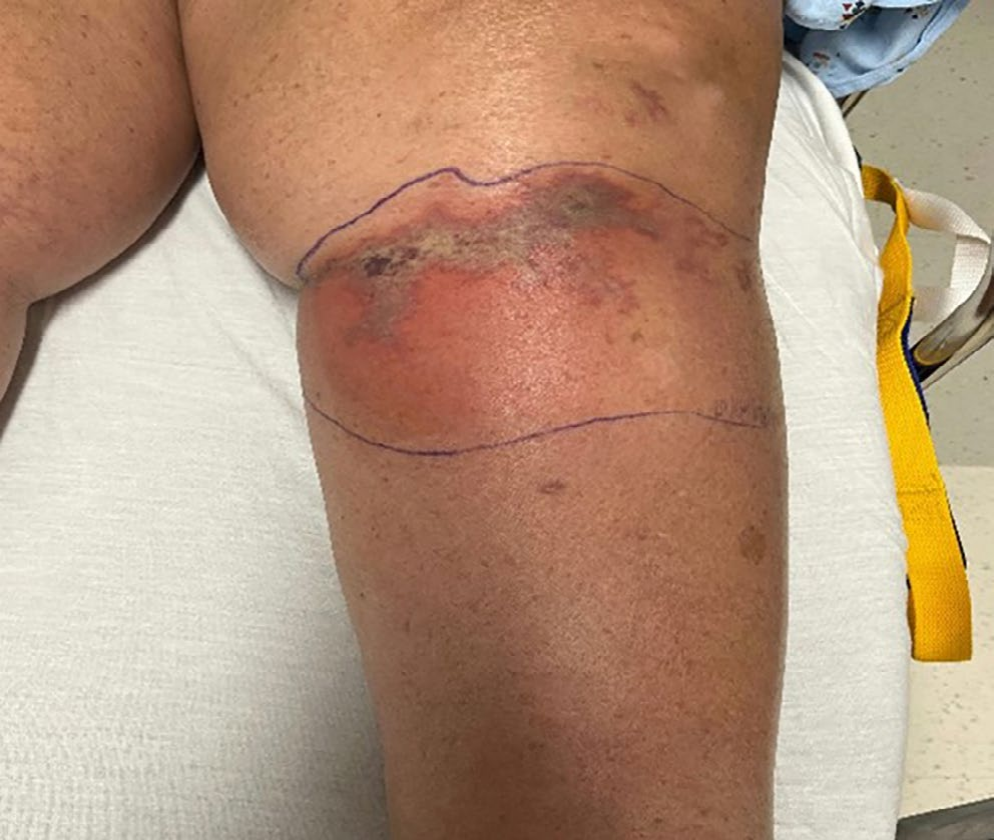
Wound at first hospitalization after phenol neurolysis. The wound appeared as an erythematous violaceous dusky patch with an overlying bulla and was characterized as a chemical reaction to phenol with possible superimposed cellulitis.

The patient pursued a second opinion at our institution on the same day. She was evaluated in our emergency department, and laboratory findings were largely unrevealing except for a C‐reactive protein level of 14 (reference, < 5.0 mg/L) and hemoglobin A_1c_ of 6.8. She was diagnosed with a chemical reaction related to phenol with a possible cellulitic component (Figure 1). The patient was prescribed doxycycline 100 mg twice daily for 10 days and discharged home.

In the following days, she returned to our institution's emergency department multiple times with complaints of worsening pain, redness, and swelling related to the chemical burn. The wound had evolved to an erythematous violaceous dusky patch with an overlying bulla. She underwent repeat computed tomography with similar results to the prior study and was referred to dermatology and vascular surgery for evaluation.

Dermatology confirmed the diagnosis of phenol‐induced chemical burn and advised wound care with the application of triamcinolone cream, petroleum jelly, and Vanicream (Pharmaceutical Specialties Inc). Vascular surgery recommended venous and arterial Doppler studies, along with computed tomography angiography of the abdomen and pelvis with lower extremity runoffs. These studies were completed and did not reveal any vascular process that could explain the patient's symptoms.

**FIGURE 2:**
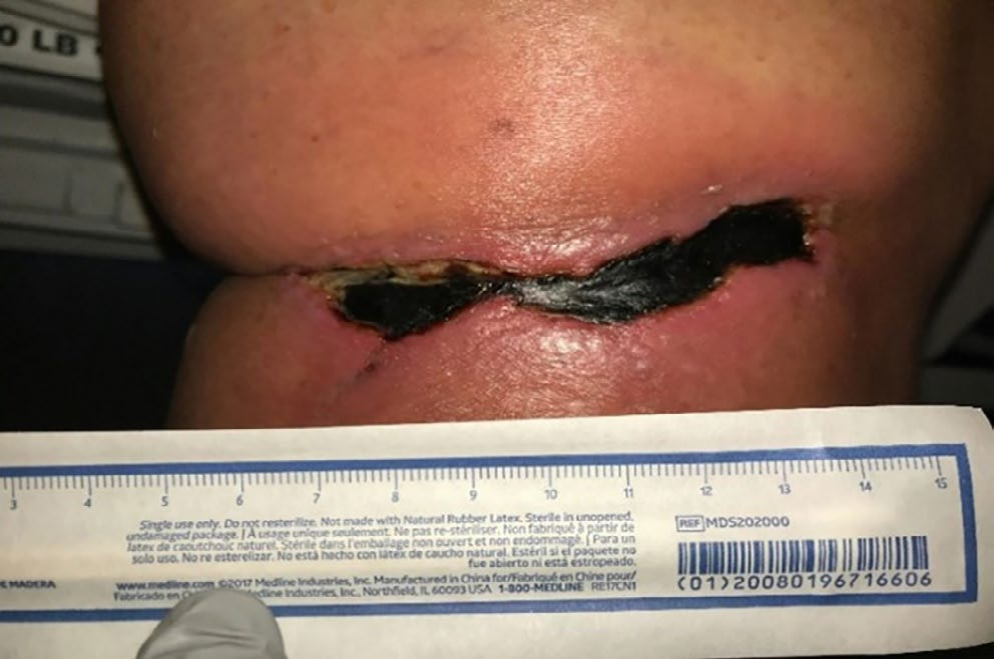
Wound approximately 3 weeks after phenol neurolysis following a 10‐day course of antibiotics and local wound care with application of triamcinolone, petroleum jelly, and Vanicream. The wound was characterized as cellulitis with a central black eschar and purulence.

After approximately 3 weeks from the initial emergency room visit at our institution, the patient developed open wounds distal to the left knee, proximal to the tibia, with a central black eschar and purulence (Figure 2). Dermatology recommended that she return to the emergency department for further evaluation. In the emergency department, arterial and venous left lower extremity ultrasounds were repeated and were again unrevealing. Magnetic resonance imaging of the left leg showed diffuse subcutaneous edema about the knee without fluid collection, abscess, or evidence of osteomyelitis. The patient was admitted to the hospital and started on empiric vancomycin and levofloxacin for suspected secondary cellulitis.

## 4 Conclusion and Results

Ultimately, our patient's chemical burn progressed to an infected ulcer that proved difficult to treat. The initial wound culture was positive for *Proteus mirabilis* , which was resistant to trimethoprim‐sulfamethoxazole, ampicillin, and tetracycline. The plastic surgery team recommended debridement by wound care; however, due to intense pain, the patient could not tolerate the procedure. She continued to receive local wound care and, per recommendation from the Division of Infectious Diseases, the patient was transitioned to a 14‐day course of amoxicillin‐clavulanate 875 mg twice daily and discharged.

**FIGURE 3:**
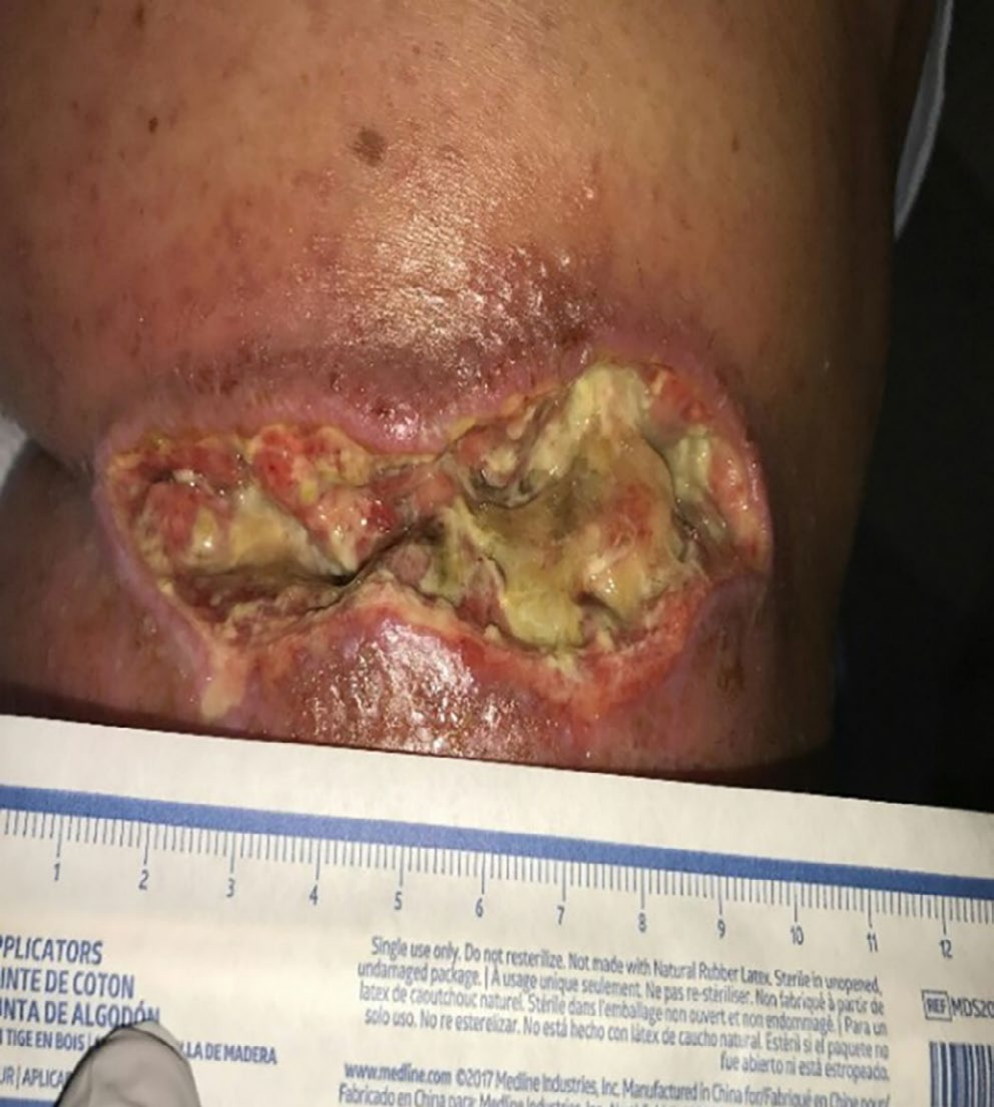
Wound approximately 6 weeks after phenol neurolysis following multiple courses of antibiotics and adherence to wound care. The wound appeared as a larger ulceration with purulent slough and was characterized as a stage 4 ulcer with nonviable fibrinous tissue.

Despite completing the full course of antibiotics and reportedly adhering to wound care instructions, the infected ulcer failed to respond to therapy and continued to progress. The patient was readmitted within 3 weeks due to enlarging ulceration with purulent slough (nonviable fibrinous tissue), now a stage 4 ulcer (Figure 3). Superficial wound cultures revealed pansensitive *Pseudomonas aeruginosa* and *P. mirabilis* resistant to ampicillin, levofloxacin, trimethoprim‐sulfamethoxazole, and tetracycline. The patient was subsequently discharged home on a prolonged course of amoxicillin‐clavulanate 875 mg twice daily and levofloxacin 750 mg/day for 28 days. Vacuum‐assisted wound closure was also performed in the wound clinic, and the wound ultimately healed.

This case report highlights the potential complications associated with phenol injection as a nerve block, particularly in patients with comorbidities such as diabetes. Our case demonstrates that tissue necrosis following phenol neurolysis can predispose diabetic patients to cellulitis, which may be challenging to treat. While phenol neurolysis remains an effective and economical treatment for chronic pain, adverse effects, though rare, underscore the importance of careful patient selection and consideration of comorbidities. Further investigation into risk factors associated with phenol‐induced tissue necrosis and cellulitis is essential to enhance the safety and efficacy of phenol neurolysis applications.

## 5 Discussion

Chemical neurolysis and radiofrequency ablation as solutions for chronic malignant pain and spasticity have been documented, especially for patients with cancer and traumatic brain injury [7]. Further, the use of chemical agents for neurolysis has become more accepted in treating patients with chronic nonmalignant pain, such as osteoarthritis [8]. Chemical neurolysis has certain advantages over spinal catheters for chronic pain management, including reduced treatment duration, admission time, bleeding risks, infection risks, and cost‐effectiveness [5]. Slatkin and Rhiner [5] demonstrated more than 50% pain reduction with a single phenol neurolysis session.

While the positive effects and successes of phenol neurolysis have been generously documented, severe tissue necrosis, ulceration, and infection as presented in our case appear to be rare consequences. Risso et al. [9] described local hematoma, pain at the injection site, swelling, and numbness as reported reactions; however, these reactions were considered minor and of short duration, with no additional reactions 1 month following the procedure. Wood [10] in her review of phenol, reported a 3% to 10% frequency of painful paresthesia and hyperesthesia, with effects noted to be temporary and lasting from 2 months to 1 year. In another study by Weksler et al. [11], the authors describe chemical neuritis, the intense burning pain along the distribution of the nerve, as a common adverse effect of phenol neurolysis; however, this adverse effect was not experienced by any of their 42 subjects. Our patient's pain and wound at the injection site persisted longer and with greater intensity than their reported reactions. In contrast, our patient did experience severe burning pain at the injection site immediately following the procedure. Her pain persisted and intensified with the subsequent development of a chemical burn that eventually progressed to a nonhealing skin ulcer.

Berry and Olszewski [1] expanded on the use of phenol injection for nerve blocks and delineated the benefits it could provide in a series of two cases. The authors further elaborated that phenol has a higher affinity to vascular tissue, and a common source of major adverse effects was thrombosis due to the migration of phenol when injected near blood vessels [1]. Although thrombosis was considered a potential etiology for our patient's left lower extremity pain, swelling, and erythema, evaluation with venous Doppler ultrasound and CT angiogram studies were negative.

This report emphasizes the importance of considering the effect of comorbid conditions on the successful administration of phenol for neurolysis. Our patient's history of smoking and diabetes with episodes of hyperglycemia may have delayed the healing process, creating a suitable environment for cellulitis to manifest. Cellulitis is a common bacterial infection of the skin and develops when a break in the skin occurs allowing normal skin flora or other bacteria to penetrate and infect the dermis and subcutaneous tissue [12]. On presentation, the skin may appear as a poorly demarcated area of erythema accompanied by edema and tenderness [12]. Conditions such as diabetes mellitus, peripheral vascular disease, and lymphedema can increase one's risk of developing cellulitis [12]. It has been found that 30% to 70% of patients with diabetes will experience a cutaneous complication in their lifetime. In addition, bacterial infections in patients with diabetes are common, with *P. aeruginosa* and staphylococcal infections being the most common [12]. This infection risk persists despite the bactericidal and fungicidal properties of phenols [5]. However, the ability of this report to confidently analyze the relationship between the patient's comorbid conditions and the adverse reaction to phenol is limited. The report demonstrates a severe adverse effect of phenol neurolysis in a patient with diabetes, and future studies should explore the potential risks among this patient population and those with other comorbid conditions. It would be prudent for providers to consider patients' comorbidities and the potential adverse effects of phenol neurolysis when assessing patients for this procedure.

## Author Contributions

**Michael D. Morodomi:** data curation, investigation, writing – original draft. **Melissa P. Cortes:** conceptualization, supervision, writing – original draft, writing – review and editing. **Michael Smerina:** conceptualization, investigation, writing – review and editing. **Tatjana Gavrancic:** conceptualization, validation, writing – review and editing. **Ricardo J. Pagan:** conceptualization, formal analysis, writing – review and editing. **Adrian G. Dumitrascu:** project administration, resources, writing – review and editing. **Aditya A. Khanijo:** writing – original draft, writing – review and editing. **Nilaa Gopikrishnan:** writing – review and editing. **Aleksandra Murawska Baptista:** resources, supervision, writing – review and editing.

## Consent

Written informed consent was obtained from the patient.

## Conflicts of Interest

The authors declare no conflicts of interest.

## Data Availability

Data sharing is not applicable to this article as no new data were created or analyzed in this study.
